# Stories of intentional action mobilise climate policy support and action intentions

**DOI:** 10.1038/s41598-021-04392-4

**Published:** 2022-01-21

**Authors:** Anandita Sabherwal, Ganga Shreedhar

**Affiliations:** 1 Department of Psychological and Behavioural Sciences and Grantham Research Institute, London School of Economics and Political Science (LSE), 63-65 Aldwych, London, WC2B 4EJ UK; 2 Department of Psychological and Behavioural Sciences, London School of Economics and Political Science (LSE), Floor 3(room 3.10) Connaught House, 63-65 Aldwych, London, WC2B 4EJ UK

**Keywords:** Psychology, Environmental social sciences, Climate-change mitigation, Climate-change policy, Psychology and behaviour

## Abstract

What makes a climate story effective? We examined if short fiction stories about everyday pro-environmental behaviours motivate climate policy support, and individual and collective climate action in a nationally representative experiment (*N* = 903 UK adults). The story featuring protagonists driven by pro-environmental intentions (i.e., the intentional environmentalist narrative) increased participants’ support for pro-climate policies and intentions to take both individual and collective pro-environmental actions, more so than did stories featuring protagonists whose pro-environmental behaviours were driven by intentions to gain social status, to protect their health, and a control story. Participants’ stronger feelings of identification with the protagonist partially explained these effects of the intentional environmentalist narrative. Results highlight that narrating intentional, rather than unintentional, pro-environmental action can enhance readers’ climate policy support and intentions to perform pro-environmental action. Therefore, the intentions driving pro-environmental action may have implications for the extent to which observes identify with the actor and take pro-environmental action themselves.

## Introduction

Stories can shape our understanding, promote cooperation and inspire action^1,2^. Despite several calls for using stories to promote public support for climate policies^3^, and understanding of science^4^, storytelling remains an underused tool in climate change communication^5^. Much of the existing climate-fiction literature is clustered under the fantasy and science fiction genres portraying heroes battling dystopian futures^5,6^. Conversely, non-fiction stories featuring “real” people often cast protagonists as victims of climate change^7^. Therefore, stories about ‘commonplace heroes’ i.e., people negotiating climate-relevant actions in their everyday lives are seldom told^5^.

The kind of stories we tell matters. The narrative structure nudges readers to engage with the trade-offs and consequences of a characters’ goals and actions by resolving conflicts that the character faces as the plot progresses over time^8^. Stories are unique in encouraging readers to take an intentional stance i.e., reflect on characters’ intentions and the mental reasoning behind their behaviour, sparking questions like, “what was her goal?” and “why did she do that?”^9^. Consequently, readers’ identification with the protagonist hinges on how the protagonist’s context, mental states, actions, and dilemmas resonate with readers. Therefore, people may find stories about ‘common place’ heroes, with everyday contexts and mental states particularly engaging.

Identifying with the protagonist of a climate fiction story may in turn inspire readers’ own beliefs and actions on climate change^6^. Prior work suggests that social identification with environmentalists can motivate individuals to take pro-environmental actions^10^. Relatedly, several studies find that people’s own pro-climate attitudes and actions are linked to the beliefs^11^ and conservation behaviours^12^ of those that they identify with, especially their proximate others (such as neighbours) and peers (such as friends and family).

Although stories about people navigating everyday environmental decisions likely resonate with readers due to their familiar context, not all commonplace heroes may be equally relatable. Characters with persistent, intrinsic pro-environmental motivations, i.e., those motivated to take actions with the intention of having a positive environmental impact, and who consistently engage in collective (e.g., rallies, petitions) and lifestyle (e.g., veganism) climate actions, can be perceived as disruptive and radical^13^, or as trying to appear more moral than they actually are^14^. They may even face do-gooder derogation i.e., their advocacy might end up demotivating others because they are seen as annoying, too moral and unrelatable^15,16^. Therefore, it is possible that characters whose actions are driven by external factors (rather than intrinsic motivation), for example, someone who takes climate actions under social pressure from their friends, may be seen as more relatable and have a lower chance of demotivating others. However, evidence also suggests that those who are intrinsically (as opposed to extrinsically) motivated are more consistent in their behaviour i.e., they are more likely to persist with a behaviour over time, even in the face of difficulty^17^. This matters because, readers identify more with coherent characters, those whose actions are consistent and driven by their motivation and goals^18^.

That said, not all intrinsic motivation is alike. An individual may be intrinsically motivated, but their actions need not be taken with the goal of having a positive environmental impact. For example, a character may follow a vegetarian diet because they are intrinsically motivated to improve their health, but their intention is not to act pro-environmentally. In fact, any pro-environmental impacts that their actions have are unintended or accidental. This contrasts with a character who follows a vegetarian diet with the goal of reducing their carbon footprint. They are intrinsically motivated *and* have pro-environmental intentions. In other words, the pro-environmental impacts of their actions are intended. More specifically, the intrinsic motivation to perform a certain action (e.g., eating vegetarian diet without being externally incentivised to do so)^17^, may or may not overlap with the intention, which is the desired impact of the behaviour (e.g., to protect environment, or one’s health)^9^. In short, although both characters are intrinsically motivated and take pro-environmental actions, only the one is *intentional* in their environmentalism i.e., wants to protect the environment. Stories are particularly interesting vehicles to investigate the differences between such characters who may perform the same action but have different motivations (intrinsic or extrinsic) and intentions because stories encourage readers to reflect on characters’ actions and speculate the intent behind these actions^9^ (i.e., take the intentional stance when analysing characters’ behaviour). Whether (and how), a character’s motivations and subsequent intentions drive others’ support for climate policy and action is unclear.

In this study, we investigate if stories featuring commonplace heroes who take the same actions to mitigate climate change but have different intentions—pro-environmental, status-seeking, or even a non-environmental such as health—impact readers’ willingness to support climate policy and take climate action. We refer to the character who is driven by intrinsic motivation and aims to protect the environment as the “intentional” environmentalist, the one who is driven by extrinsic motivation and aims to gain social approval as the “status-seeking” environmentalist, and the one who is driven by intrinsic motivation that is unrelated to the environment as the “accidental” environmentalist. In doing so, we explore how the protagonists’ behaviours and mental states, integral components of character development in stories, can be portrayed so that protagonists serve as role-models for climate action while being relatable to readers.

## Methods

All experiments (pilot and main Study) were in accordance with guidelines of the Helsinki declaration and approved by the London School of Economics and Political Science (LSE) Research Ethics committee. The Study was pre-registered here. Stimuli, data, and analysis script are available here.

### Narrative development

Before conducting the experiment, we adopted an iterative, mixed-methods approach to develop treatment stories. First, we circulated the stories among behavioural science post graduate students (*N* = 30) and conducted focus group discussion to seek their feedback on the stories as well as the question, “What makes a good story?”. Themes like identifiable characters, a realistic setting, indication of the characters’ mental states, and a narrative structure that underscores the process of conflict resolution emerged. Next, in a pilot study of 500 UK adults recruited via Prolific Academic, we randomly assigned participants to read one of the four treatment stories and asked them free-response questions about their feelings towards the protagonist and attributions of the protagonist’s primary motive.

Our final story was a narration of the protagonist’s day-in-the-life. This format allowed us to present the protagonist as relatable (e.g., he binges Black Mirror; see SI) and afforded ecological validity since many social media personalities convey their personal stories using the day-in-the-life paradigm. In the three experimental conditions, participants read a story about a day in the life of the protagonist who took the same individual and collective climate actions (i.e., chose a vegan meal during lunch with friends and singed a climate petition at work), but had differing intentions—to protect the environment, to gain appreciation from others, or to improve his personal health respectively. Since we held actions constant and only varied intentions, we refer to these stories as the intentional, status-seeking, and accidental environmentalist respectively. In the control condition, participants read an unrelated story which followed the same day-in-the-life format but did not feature climate actions. 84% participants could correctly attribute the protagonist’s intentions, indicating that our story was largely successful in conveying mental states.

### Participants and procedure

**Figure 1:**
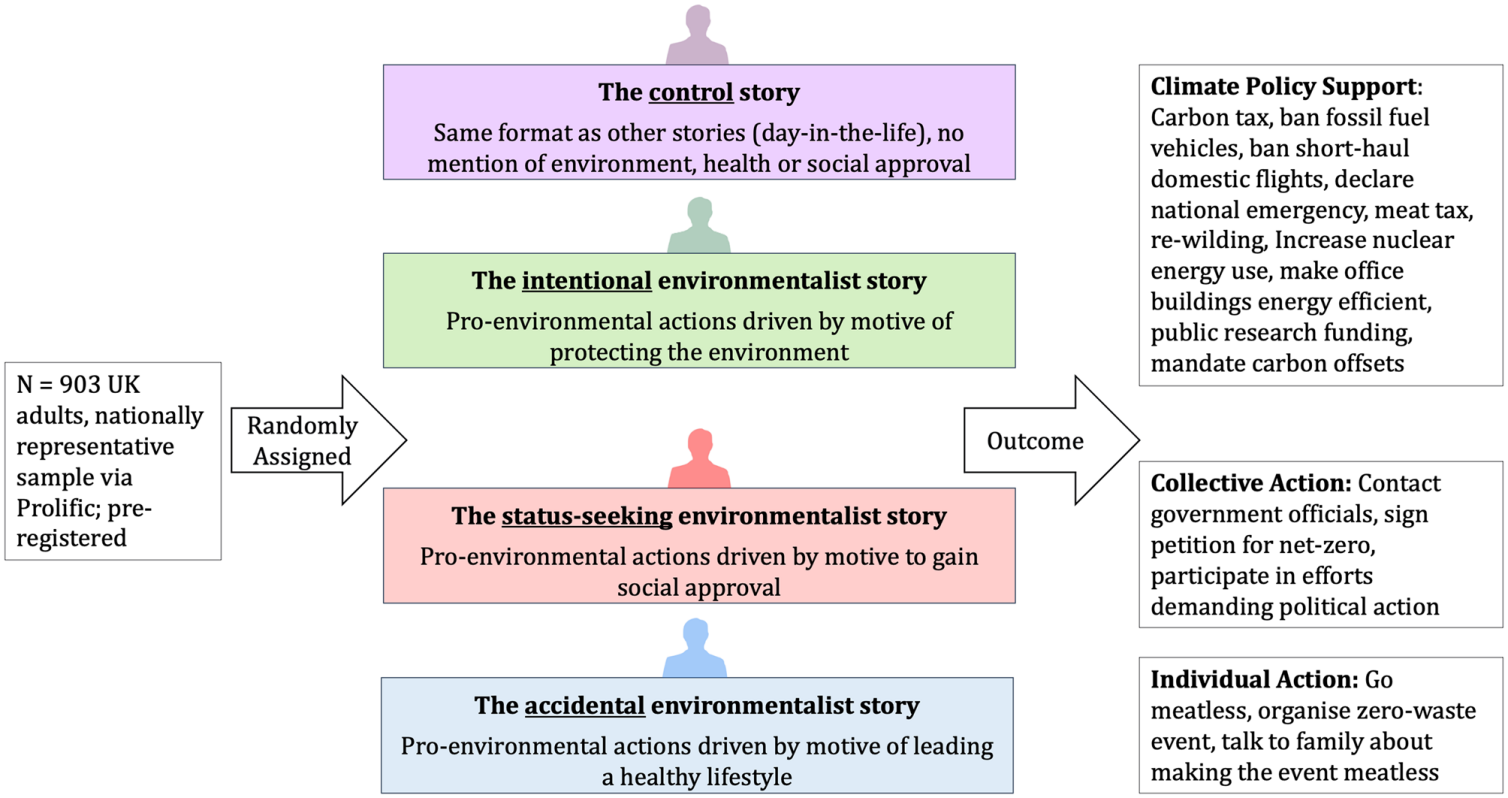
Experimental set-up. The sample was nationally representative of the UK with regards to age, gender and ethnicity; recruited via Prolific Academic; randomly assigned to one of three experimental conditions or control. Figure created by first author using Microsoft PowerPoint Version 16.54 https://www.microsoft.com/en-us/microsoft-365/powerpoint.

For the present nationally representative, pre-registered, between-subjects study, we recruited 903 UK adults via prolific academic (see SI for socio-demographic characteristics of the participant sample) to obtain more then 80% power of detecting a small-to-medium effect both in a one-way ANOVA with four groups, and in a bias-corrected mediation analysis. Upon providing informed consent, participants were randomly assigned to read one of four stories—The intentional environmentalist, status-seeking environmentalist, accidental environmentalist, or control respectively. See Fig. 1 for experimental protocol. As attention checks, participants then answered two factual questions about the story. Since All 903 participants answered at least one attention-check question correctly, no participants were excluded from our analyses (see SI for a priori criteria).

Participants then reported the extent to which they related with the protagonist on a 3-item scale (α = 0.82) that has been used in prior narrative research^6^. Next, participants reported their support for 10 climate policies (α = 0.87). These policies were selected from a government-commissioned report on the policies that typically receive low public consensus but that must be adopted for the UK to meet its long-term target of Net-Zero. Therefore, we were able to test whether our treatment narrative could solve the real-world problem of motivating support on crucial climate policies. Participants also reported how likely they were to take three collective actions (α = 0.86). To assess if, like the protagonist, participants became more motivated to take individual actions even in the face of social barriers, we asked them to rate how likely they were to take three individual actions (α = 0.80) in a social setting (family picnic). Participants also answered questions about the effectiveness of the story’s protagonist (See SI). Finally, participants provided demographic information and were debriefed.

### Measures

Measures of primary interest are listed below. A comprehensive list of other measures can be found in the SI. The following were the measured on 7-point Likert scales (1 = not at all to 7 = very much). Table 1 provides descriptive statistics and scale reliabilities for all outcome variables.

**Table 1:** Descriptive statistics of dependent variables (sample and condition).

Variable	Sample mean (SD)	Scale reliability alpha [95% CI]	Control mean (SD)	Intentional mean (SD)	Status-seeking mean (SD)	Accidental mean (SD)
Policy support	4.32 (1.15)	0.87 [0.86, 0.88]	4.22 (1.12)	4.53 (1.15)	4.23 (1.17)	4.31 (1.14)
Collective action intention	3.51 (1.59)	0.86 [0.84, 0.87]	3.38 (1.56)	3.76 (1.58)	3.52 (1.65)	3.39 (1.55)
Individual action intention	3.46 (1.65)	0.80 [0.77, 0.82]	3.33 (1.68)	3.67(1.62)	3.38 (1.64)	3.48 (1.63)
Identification	4.16 (1.31)	0.80 [0.82, 0.84]	4.08 (1.19)	4.45 (1.32)	3.76 (1.33)	4.33 (1.28)
Donation	0.48 (0.37)	NA	0.46 (0.38)	0.48 (0.36)	0.49 (0.36)	0.47 (0.37)

**Policy Support** was measured using 10 items asking participants’ support for: carbon tax, banning fossil-fuel operated cars, banning short-distance domestic flights, declaring national climate emergency, meat tax, rewilding, nuclear energy, renovating office buildings for energy efficiency, invest in sustainable aviation fuel research, mandatory carbon offsets for flight tickets (Scale M = 4.32, SD = 1.15, α = 0.87). These items were derived from policies recommended by UK Committee on Climate Change’s report: The sixth carbon budget, the UK’s path to net zero^19^.

**Intentions to take public action at the collective level (collective action intentions)** were assessed using three items asking the extent to which participants would be willing to: participate in efforts demanding political climate action, contact government officials to demand action, and sign a net-zero petition (Scale M = 3.51, SD = 1.59, α = 0.86). Items were adapted from prior work on collective climate action^20,21^. This scale measured participants’ intentions to take action alongside others in a public sphere (such as in protests and political fora)^22^.

**Intentions to take action in every-day life, at the individual level (Individual action intentions)** were measured by giving participants a scenario in which they were to organise a family barbecue picnic and asking the extent to which they would be willing to take 3 actions: talk to their family about organising a meatless picnic, make the picnic zero-waste, themselves go meatless for the picnic (Scale M = 3.46, SD = 1.65, α = 0.80). This scale assessed participants’ intentions to make on-going changes to their lifestyle. Though taken at the individual level, such action could be conceptualised as contributing to the collective cause of mitigating climate change^22^.

**Identification with character:** Was measured using three items asking the extent to which participants: felt the emotions George (the protagonist) was feeling, imagined what it would be like to be in George’s position, think that George was like their friends and family (Scale M = 4.16, SD = 1.31, α = 0.80). Adapted from prior research on fiction narratives^6^, the identification scale was able to measure the extent to which participants could resonate with George’s mental states and the extent to which they found him to be socially proximate (like friends and family), both of which are aspects of identification that can enhance individuals’ pro-climate attitudes and beliefs^6,11,12^.

**Donation amount:** Participants were asked how much of their income from the experiment (1 GBP) they would be willing to donate to a pro-environmental charity of their choice (M = 0.48, SD = 0.37). To offer an adequate incentive, they were told that 10% of participants would be selected at random, their income from the experiment and stated donation amount will be multiplied by 10 and allocated between the participant and the charity they have indicated as per their preference stated here. This was a modified charitable giving task adapted from prior research on narratives to promote conservation^23^. Participants could choose from a variety of charities to avoid any confounding effects of their preferred environmental action. Research in experimental economics shows that providing incentives to only a subset of participants (such as by providing a 10% chance that their income will be multiped and stated amount donated) reduces cost and experimenters’ efforts while producing results comparable with providing incentives to all participants (e.g., multiplying every participants’ income and donating their stated amount)^24^. Although we endeavoured to create a realistic donation task providing diverse options of charities and eliminating income-effects, we cannot empirically rule out that the design of our donation measure could have impacted participants’ donation.

## Results

**Figure 2:**
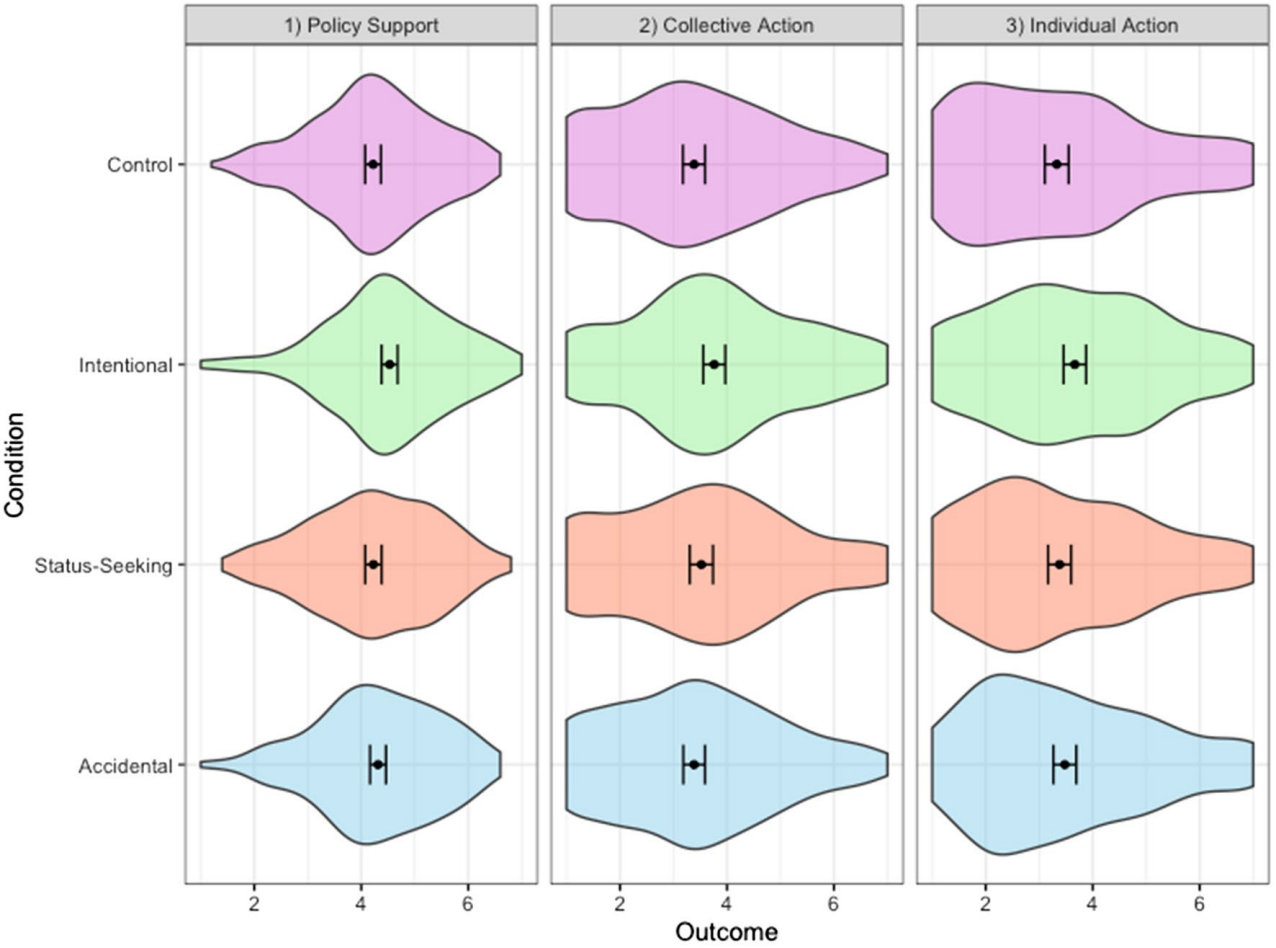
Climate policy support and action intentions across stories. Error bars represent 95% confidence interval of the means. Policy support, collective action and individual action intentions measured using 10-item, 3-item and 3-item composites respectively. All items measured on Likert scales of 1(not at all) to 7(extremely).

**Figure 3:**
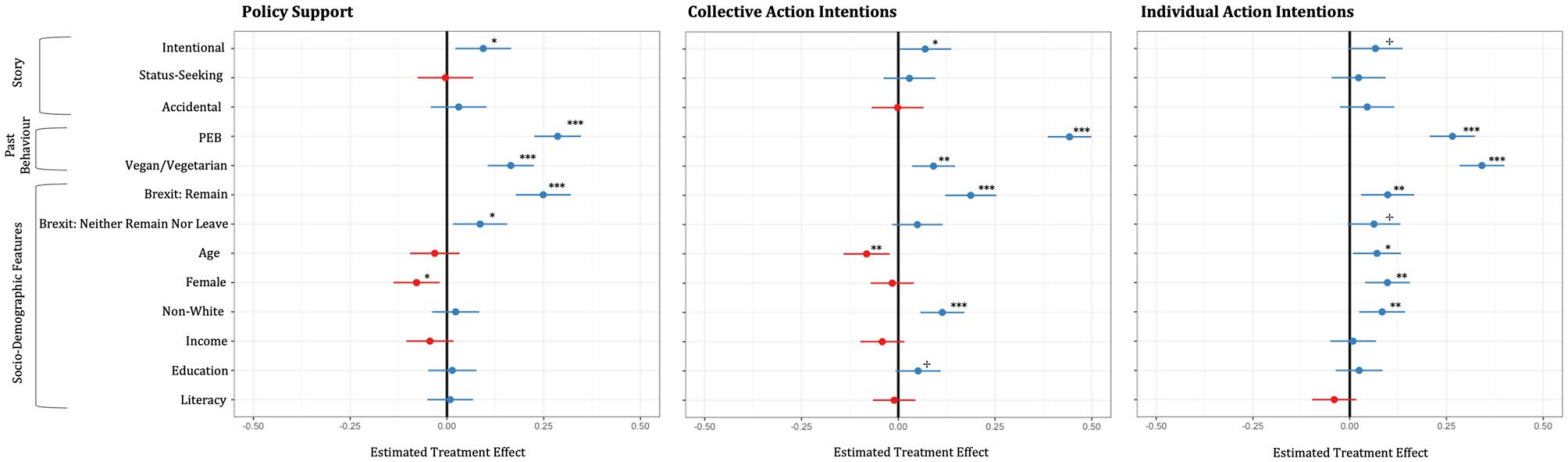
Effect of stories on climate policy support and action intentions. ^†^*p* < 0.1, *p < 0.05, **p < 0.01, ***p < 0.001. Estimates are standardised coefficients. Error bars represent 95% confidence. Blue and red represent positive and negative effects respectively. All outcomes measured using composites, on Likert scales of 1(not at all) to 7(extremely). Omitted categories: Control group, non-vegetarian/vegan diet, Brexit: leave, male, white. Scales for ordinal/numerical variables: PEB (Past environmental behaviour)—0–2 scale, Age—continuous numerical variable, Income—Likert scale of 1(less than £20,000) to 6 (more than £100,000), Education—Likert scale of 1(less than O level) to 10 (Doctorate or other professional degree), Literacy—Likert scale of 1 (no proficiency) to 6 (native/bilingual proficiency).

Does the type of story affect climate policy support? As shown in Fig. 2, those who read the intentional environmentalist story reported greater support for climate policies (M = 4.53, SD = 1.15), relative to those who read the control (M = 4.22, SD = 1.22; *t*(448) = 2.89, *p* = 0.004, *d* = 0.27, 95% CI [0.09, 0.46]), status-seeking environmentalist (M = 4.23, SD = 1.17; *t*(452) = 2.78, *p* = 0.006, *d* = 0.26, 95% CI [0.08, 0.45]) and accidental environmentalist (M = 4.31, SD = 1.14; *t*(453) = 2.02, *p* = 0.04, *d* = 0.19, 95% CI [0.004, 0.37]) stories. This effect persisted when we controlled for factors such as past behaviour and socio-demographic characteristics (Fig. 3). There were no significant differences in socio-demographic factors across conditions (See SI). The accidental and status-seeking environmentalist stories failed to enhance policy support either when compared to the control, or to one another (See SI for pairwise comparisons).

We also tested if the stories motivated participants to take public climate action with others (what we refer to as collective action) and in their daily lives at an individual level, with the understanding that it may contribute to a collective cause (what we refer to as individual action). Those who read the intentional environmentalist story reported greater intentions of taking both collective (M = 3.76, SD = 1.58) and individual climate actions (M = 3.67, SD = 1.62), relative to those in the control condition (M_collective_ = 3.38, SD_collective_ = 1.56; M_Individual_ = 3.33, SD_Individual_ = 1.68; Collective action: *t*(448) = 2.56, *p* = 0.01, *d* = 0.24, 95% CI [0.05, 0.43]; Individual action: *t*(446) = 2.17, *p* = 0.03, *d* = 0.20, 95% CI [0.02, 0.39]). Neither the status-seeking environmentalist (M_collective_ = 3.52, SD_collective_ = 1.65; M_Individual_ = 3.38, SD_Individual_ = 1.64), nor the accidental environmentalist (M_collective_ = 3.39, SD_collective_ = 1.55; M_Individual_ = 3.48, SD_Individual_ = 1.63) stories enhanced collective and individual action intentions, relative to the control and one another. Moreover, participants in the intentional environmentalist condition reported stronger collective action intentions than those in the accidental environmentalist condition *t*(453) = 2.56, *p* = 0.01, *d* = 0.24, 95% CI [0.05, 0.42], and marginally significantly stronger individual action intentions than those in the status-seeking environmentalist condition (See SI)*.*

What might be driving the impact of the intentional environmentalist story? Consistent with past literature on the role of social identification in driving pro-environmental attitudes^6,10^, we found that participants’ identification with the protagonist partially mediated the effect of the intentional environmentalist narrative on their policy support and action intentions. Participants who read the intentional environmentalist story identified more strongly with the protagonist than did those in the control condition (*b* = 0.36, *SE* = 0.12, *t* = 3.02, *p* = 0.003). Identification with the protagonist in turn predicted participants’ policy support (indirect effect: b = 0.11, SE_boot_ = 0.04, 95%Boot CI [0.04, 0.19]), collective (indirect effect: b = 0.14, SE_boot_ = 0.05, 95%Boot CI [0.04, 0.25]) and individual action intentions(indirect effect: b = 0.14, SE_boot_ = 0.05, 95%Boot CI [0.05, 0.24]). Similar mediation patterns emerged when we compared the intentional environmentalist with the status-seeking and accidental environmentalist stories (See SI for detailed mediation analyses).

We found no main effect of condition on the amount participants chose to donate to environmental charities, F (3,899) = 0.41, MS = 0.06, *p* = 0.747 (See SI for details).

## Discussion

Our study shows climate fiction need not be dystopian or catastrophic to mobilise action. Short fiction stories featuring commonplace heroes taking intentional pro-environmental action in everyday contexts like social lunches and at work can encourage readers’ climate policy support and action intentions. The benefits of intrinsically motivated pro-environmental behaviour have been debated^25^ as has whether an action’s consequences is more important than the motives behind the actions^26^. All stories we tested featured commonplace heroes taking the same climate actions, yet only protagonists driven by pro-environmental intentions induced stronger support for climate policy and climate action intentions among readers. Our findings show that the intentions driving pro-environmental actions can act as crucial social learning cues in stories and therefore, cannot be dismissed.

Stories can prompt readers to attribute intentions to characters^9^ and speculate what mental states caused their actions^27^. Intentions are indicative of motivation and instrumental for behaviour^28^. Intrinsically and extrinsically motivated behaviour is performed because the activity itself provides satisfaction or to achieve an unrelated, external goal respectively^29^. So, the same pro-environmental action can either be intrinsically or extrinsically motivated based on whether the actor performs it to protect the environment (i.e., has pro-environmental intentions) or to gain social approval (i.e., has status-seeking intentions) respectively. As a result, intentions are crucial social signals in narratives. Readers expect characters to take actions consistent with their intentions^9^ and therefore use intentions to infer if the actor is intrinsically or extrinsically motivated, form moral judgements about the character^27^, infer their goals and values, and predict their future behaviour.

So, if we know an actor intends to protect the environment, we expect them to behave (e.g., go meatless) in accordance with their goals (e.g., to limit climate change), and to espouse consistent values (e.g., environmentalism)^30^.

Narrating intentional states may also enable readers to experience their own lives in ways that match the protagonist’s fictional life, and identification with the protagonist may encourage readers to adopt similar pro-environmental values and goals. Related research has found that connecting pro-environmental actions with intrinsic motivation, values and identity can encourage climate policy support and catalyse positive pro-environmental behavioural spill-overs^31,32^. Personal stories of intentional pro-environmental action may prompt readers to reflect on their own pro-environmental motivations and values, and could therefore be explored as a novel tool to address the pesky challenge of motivating climate policy support across different domains.

Although the intentional environmentalist narrative increased support for climate policy and intentions to take collective and individual pro-environmental actions, it did not increase the amount participants donated to environmental charities, relative to the control. This finding is consistent with other research that finds the effects of narratives on pro-environmental self-reported attitudes, beliefs, and policy support, but limited effects on revealed donations behaviour^6,7,23^. Does this mean that narratives are unable to change pro-environmental behaviour? Not necessarily: it is possible that our treatment may have impacted pro-environmental behaviours which are more congruent with the narrative (e.g. dietary choices), but which were not measured in our experiment. Indeed, while many studies use donation outcomes as a proxy for pro-environmental behaviour, recent experimental evidence shows little to weak correlations between donation behaviour elicited using incentivised tasks to helping and pro-social behaviours in the field^33^. This is largely because the determinants of behaviours vary greatly based on situational context.

That said, the absence of effect on our donation measure, though possibly an artifact of the experimental setup, raises an important question: Can stories change behaviour? It is possible that narrative effect behaviour through factors like how immersive the storytelling medium is, as well as how often people are exposed to the story^34,35^. For example, TV and Radio narratives are more effective in shaping health behaviours than are print narratives^34^. It may also be the case that that pro-environmental behaviour change is a process, in which attitude change (which, in our study narratives are able to accomplish) needs to be combined with repeated exposure to messages over time. Indeed, much psychological research shows that triggering affect, subjective norms, attitudes and intentions are important precedents (and predictors) of behaviour change^28,36^. Therefore, by enhancing readers’ intentions to act and support climate policies, the intentional environmentalist narrative in our study may have sparked the behaviour change process that can be further accelerated by narrating the story through immersive media (like videos or virtual reality), repeated exposure to the narrative (i.e., increasing dosage), and even encouraging people to reflect on the story (i.e., increase engagement).

Our findings show that narratives can be crucial tools to motivate policy support and begin the process of behaviour change. These findings have implications for designing climate advocacy and campaigns. Advocates, including members of the public, artists, writers, and policymakers, can utilise storytelling to not only narrate climate actions, but also convey the mental states and values that drive it. Based on our findings, we expect those advocates—both real and fictional—whose actions are accompanied by cues of their pro-environmental intentions may be more effective in motivating climate policy support and actions intentions, compared to those whose actions appear to be driven by a desire to gain social-approval, or another unrelated cause. Of course, good intentions are insufficient by themselves and must also engender impactful behaviour. Research on the intention-behaviour gap^37^ shows that intentions may not always lead to behaviour. As a result, narratives may serve as one element of a multi-pronged approach to motivating consistent pro-environmental behaviour and policy support—they may spark intentions which other tools (like repeated exposure to persuasive messages) can turn into real action.

Future research can build on this work by investigating how to bridge the intention-behaviour gap and harness intentions for sustained climate action, studying why characters’ mental states impact readers’ identification with protagonist, and comparing the effects of personal narratives with other popular climate-fiction stories^6,7^. Research can also more closely investigate the process and circumstances through which narratives can change behaviour, identifying factors that mediate and moderate this effect like repeated exposure and the storytelling medium.

## Supplementary Information

Supplementary Information.

## Data Availability

The Study was pre-registered at https://osf.io/y9s6m?view_only=4c4b610aba114875a47ac8af5bcbedfc. Stimuli, data and analysis script are available at https://osf.io/xqrsh/?view_only=79e5ce003c094cfc95f5c6e21b818be8.
