# Supplementary material for: Stories of intentional action mobilise climate policy support and action intentions
Source: Sci Rep. 2022 Jan 21;12:1179. doi: 10.1038/s41598-021-04392-4 (PMC8782940; doi:10.1038/s41598-021-04392-4)
Supplement: Supplementary file 1 — Supplementary Information. [file 41598_2021_4392_MOESM1_ESM.docx]

**Supplementary Materials**

INDEX

EXPERIMENTAL SET UP 2

PRE-REGISTRATION 2

EXPERIMENTAL DESIGN 2

STIMULI & DATA 2

SAMPLE CHARACTERISTICS AND DESCRIPTIVE STATISTICS 2

LIST OF ITEMS AND DESCRIPTIVE STATISTICS 5

ANALYTIC PROCEDURE 6

EFFECTS ON POLICY SUPPORT 7

ANOVA AND PAIRWISE COMPARISONS 7

REGRESSION MODEL INCLUDING COVARIATES 7

EXPLORATORY MEDIATION ANALYSES 8

EFFECTS ON COLLECTIVE ACTION INTENTIONS 8

ANOVA AND PAIRWISE COMPARISONS 8

REGRESSION MODEL INCLUDING COVARIATES 9

EXPLORATORY MEDIATION ANALYSES 9

EFFECTS ON INDIVIDUAL ACTION INTENTIONS 10

ANOVA AND PAIRWISE COMPARISONS 10

REGRESSION MODEL INCLUDING COVARIATES 11

EXPLORATORY MEDIATION ANALYSES 12

EFFECTS ON PERCEIVED EFFECTIVENESS OF CANDIDATE 12

ANOVA AND PAIRWISE COMPARISONS 12

REGRESSION MODEL INCLUDING COVARIATES 13

EXPLORATORY MEDIATION ANALYSES 13

EFFECTS ON DONATIOn 14

ANOVA AND PAIRWISE COMPARISONS 14

DEVIATION FROM PRE-REGISTRATION 15

References 15

1. **Experimental Set-up:**

**1.1 The study was pre-registered at:** <https://osf.io/y9s6m/?view_only=4c4b610aba114875a47ac8af5bcbedfc>

**1.3** **Experimental design** is depicted in Figure S1

**Figure S1: Experimental design**


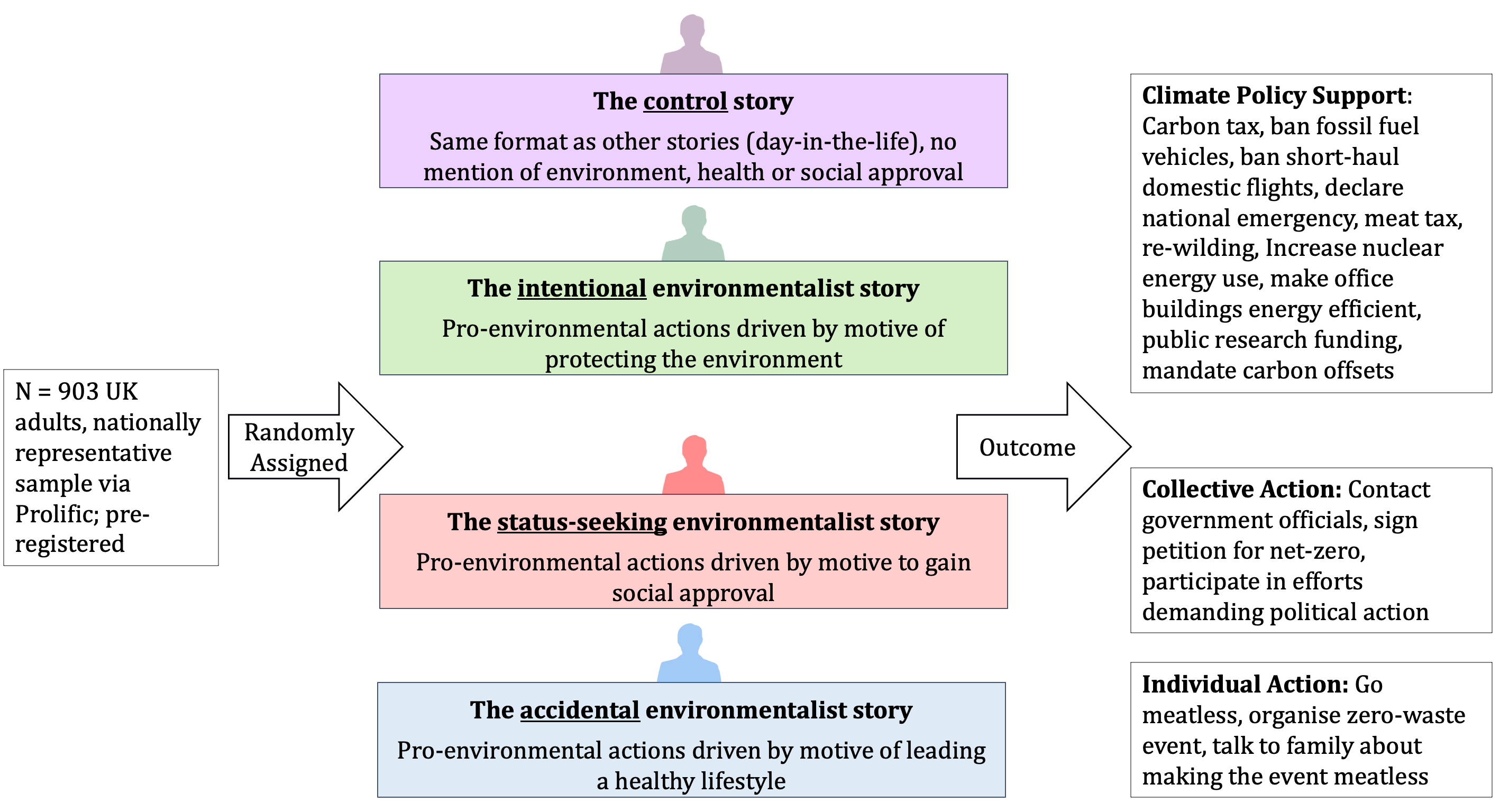


Note: (i) Pre-registered online study with a nationally representative sample (N = 903) recruited via Prolific Academic. (ii) Stories in all conditions followed the same format, describing a day in the protagonist’s life, where he takes the same pro-environmental actions in all conditions. Figure created by first author using Microsoft PowerPoint Version 16.54 <https://www.microsoft.com/en-us/microsoft-365/powerpoint>.

**1.4 Stimuli** i.e., the stories that participants read in each condition, anonymised data files and analysis script can be found on this link: <https://osf.io/xqrsh/?view_only=79e5ce003c094cfc95f5c6e21b818be8> . All stories were categorised as “fairly easy to read” based on the Flesch Reading Ease score^1^.

1. **Sample Characteristics (bullet points, one per each variable + no significant effect of condition)**
   1. **Sample size:** We recruited enough participants to obtain at least 80% chance (power) of detecting a small-to-medium effect size. Our primary analysis involved a one-way between subjects ANOVA. 500 participants are required to achieve 0.80 power in a one-way between-subjects ANOVA with the effect size of 0.15 (f = 0.15, 1- ß = 0.80, α= 0.05, number of groups = 4). One of exploratory hypotheses also involved mediation. A sample size of 400 (i.e., 200 per group) is required to achieve 0.80 power of detecting a small-medium effect (α path = 0.14, ß path = 0.39) in a Bias corrected mediation analysis (Fritz & Mackinnon, 2007). Factoring in the possibility that around 10% may fail attention and a seriousness check, we recruited 903 participants to have 200+ participants in each group. All participants passed our a-priori attention check by answering at least one of two factual questions and the seriousness check question correctly. Therefore, our final sample consisted of 903 UK adults.
   2. **National Representation:** To ensure our sample reflected the UK population, we recruited a nationally representative population (based on age, ethnicity and gender) via prolific academic. Participants were randomly assigned to one of the four conditions at the start of the survey.
   3. **Gender**: The sample consisted of 440 male, 459 female and 4 other/non-binary identifying participants. The gender distribution across conditions was:
      1. Control: 114 male, 108 female
      2. Intentional environmentalist: 97 male, 129 female, 2 other/non-binary
      3. Status-seeking environmentalist: 114 male, 111 female, 1 other/non-binary
      4. Accidental environmentalist: 115 male, 111 female, 1 other/non-binary
   4. **Ethnicity**: The ethnic composition of the sample was:
      1. 755 White (177 in control, 192 in intentional, 193 in status-seeking, and 193 in accidental environmentalist conditions)
      2. 37 Black, African, Caribbean or Black British (12 in control, 12 in intentional, 6 in status-seeking, and 7 in accidental environmentalist conditions)
      3. 79 Asian or Asian British (17 in control, 18 in intentional, 21 in status-seeking, and 23 in accidental environmentalist conditions)
      4. 21 Mixed or Multiple ethnic groups (10 in control, 3 in intentional, 6 in status-seeking, and 2 in accidental environmentalist conditions)
      5. 11 other ethnic group participants. (6 in control, 2 in intentional, 0 in status-seeking, and 3 in accidental environmentalist conditions)
   5. **Political Party Affiliation:** The political party support across the sample was:
      1. 343 supported Labour (72 in control, 90 in Intentional, 91 in status-seeking and 90 in accidental environmentalist condition)
      2. 287 supported Conservatives (82 in control, 71 in Intentional, 71 in status-seeking and 63 in accidental environmentalist condition)
      3. 26 supported Scottish National Party (1 in control, 7 in Intentional, 10 in status-seeking and 8 in accidental environmentalist condition)
      4. 107 supported Liberal Democrats (34 in control, 30 in Intentional, 22 in status-seeking and 21 in accidental environmentalist condition)
      5. 78 supported Green Party (18 in control, 15 in Intentional, 21 in status-seeking and 24 in accidental environmentalist condition)
      6. 29 supported UK Independence Party (8 in control, 5 in Intentional, 7 in status-seeking and 9 in accidental environmentalist condition)
      7. 33 reported other/no party affiliation (7 in control, 10 in Intentional, 4 in status-seeking and 12 in accidental environmentalist condition)
   6. **Dietary preference:** Participants’ dietary preferences were:
      1. 561 Omnivore (146 in control, 130 in Intentional, 143 in status-seeking and 142 in accidental environmentalist condition)
      2. 48 Pescatarian (9 in control, 13 in Intentional, 16 in status-seeking and 10 in accidental environmentalist condition)
      3. 190 Flexitarian (40 in control, 54 in Intentional, 45 in status-seeking and 51 in accidental environmentalist condition)
      4. 52 Vegetarian (14 in control, 13 in Intentional, 11 in status-seeking and 14 in accidental environmentalist condition)
      5. 25 Vegan (7 in control, 8 in Intentional, 5 in status-seeking and 5 in accidental environmentalist condition)
      6. Collapsing these categories, there were 77 participants who were vegetarian/vegan i.e., did not eat meat (21 in control, 21 in Intentional, 16 in status-seeking and 19 in accidental environmentalist condition) and 826 participants who ate meat (201 in control, 207 in Intentional, 210 in status-seeking and 208 in accidental environmentalist condition)
   7. **Past Environmental Behaviour (PEB):** To measure past environmental behaviour, participants were asked if they have donated their money or time to an environmental cause (1=yes, 0=no) and if they have demanded that greater action be taken on climate change by signing a petition and/or contacting their MP (1=yes, 0=no). Participants’ final PEB was derived by adding their responses to both these items. A one-way ANOVA showed that PEB did not differ significantly by condition F (3,899) =1.37, MS=0.79, *p*=0.25. The overall mean and mean (SD) for each condition are present in Table S1.
   8. **Age**: A one-way ANOVA showed that age did not differ significantly by condition F (3,899) =0.87, MS=215.73, *p*=0.46
   9. **Brexit support**: Brexit support was measured on a 3-point scale (1= Leaver, 2=Neither, 3=Remainer). A one-way ANOVA showed that Brexit support did not differ significantly by condition F (3,899) =0.10, MS=0.08, *p*=0.96
   10. **Income**: Income was measured on a likert scale (1= less than £20,000, 2=£20,001-£40,000, 3=£40,001-£60,000, 4=£60,001-£80,000; 5=£80,001-£100,000, 6= more than £100,000). A one-way ANOVA showed that Income did not differ significantly by condition F (3,899) =1.60, MS=1.60, *p*=0.19
   11. **Education**: Education was measured on likert scale ( 1="Less than O Level", 2="O Level", 3="A Level", 4="Some college but left before graduating", 5="Currently in college", 6="Certificate of Higher Education", 7="Diploma of Higher Education", 8="Bachelor's degree", 9="Master's degree", 10="Doctorate or other professional qualification")

A one-way ANOVA showed that level of education did not differ significantly by condition F (3,899) =0.22, MS=1.68, *p*=0.88

- 1. **Reading Literacy**: Was self-reported on a likert scale (1 = “No proficiency", 2 = “Elementary proficiency", 3 = “Limited working proficiency", 4 = “Professional working proficiency",5 = “Full working proficiency",6 =“ Native / bilingual proficiency"). A one-way ANOVA showed that reading literacy did not differ significantly by condition F (3,899) =0.65, MS=0.11, *p*=0.59

**Table S1: Descriptive statistics of covariates**

| Variable | Sample Mean(SD) | Control Mean(SD) | Intentional Mean(SD) | Status-Seeking Mean(SD) | Accidental Mean(SD) | Difference across conditions (p value) |
| --- | --- | --- | --- | --- | --- | --- |
| PEB | 0.62(0.76) | 0.57(0.74) | 0.70(0.81) | 0.58(0.72) | 0.64(0.76) | 0.25 |
| Age | 47.02(15.76) | 47.83(15.39) | 47.69(15.91) | 45.70(15.62) | 46.89(16.12) | 0.46 |
| Brexit Support | 2.27(0.89) | 2.24(0.90) | 2.89(0.90) | 2.27(0.88) | 2.69(0.89) | 0.96 |
| Income | 1.91(1.00) | 1.96(0.99) | 1.85(0.99) | 2.00(1.08) | 1.83(0.96) | 0.19 |
| Education | 6.25(2.75) | 6.32(2.71) | 6.15(2.81) | 6.33(2.68) | 6.10(2.81) | 0.88 |
| Reading Literacy | 5.87(0.40) | 5.90(0.34) | 5.85(0.40) | 5.88(0.47) | 5.86(0.39) | 0.59 |

Note: Includes covariates that were measured as numerical variables. PEB refers to Past Environmental Behaviour. Difference across condition analysed using one-way ANOVA

1. **Items Used to measure each Outcome Variable**

NOTE: All items except for donation amount were measured on 7-point likert scales (1=not at all to 7=very much). Table S2 provides descriptive statistics and scale reliabilities for all outcome variables.

**3.1 Policy Support** was measured using 10 items asking participants’ support for: carbon tax, banning fossil-fuel operated cars, banning short-distance domestic flights, declaring national climate emergency, meat tax, rewilding, nuclear energy, renovating office buildings for energy efficiency, invest in sustainable aviation fuel research, mandatory carbon offsets for flight tickets. These items were derived from policies recommended by UK Committee on Climate Change’s report: The sixth carbon budget, the UK’s path to net zero^2^.

**3.2** **Collective Action Intention** was assessed using three items asking the extent to which participants would be willing to: participate in efforts demanding political climate action, contact government officials to demand action, and sign a net-zero petition. Items adapted from prior work on collective climate action^3,4^.

**3.3 Individual Action Intentions** were measured by giving participants a scenario in which they were to organise a family barbecue picnic and asking the extent to which they would be willing to take 3 actions: talk to their family about organise a meatless picnic, make the picnic zero-waste, themselves go meatless for the picnic.

**3.4 Perceived Effectiveness of Character:** To assess how effective George, the story’s protagonist, would be as a climate advocate, we asked participants how much they were interested in learning from George about climate change, willing to trust the information George provides about climate change, and join a climate campaign if George asked them to.

**3.5 Donation Amount:** Participants were asked how much of their income from the experiment (1 GBP) they would be willing to donate to a pro-environmental charity of their choice. They were told that a few participants would be selected at random, their income from the experiment and stated donation amount will be multiplied by 10 and allocated between the participant and charity as per their preference stated here.

**3.6 Identification with Character:** Was measured using three items asking the extent to which participants: felt the emotions George was feeling, imagined what it would be like to be in George’s position, think that George was similar to their friends and family

**Table S2: Descriptive Statistics of Dependent Variables (Sample and Condition)**

| Variable | Sample Mean (SD) | Scale Reliability Alpha [95%CILL, 95%CIUL] | Control Mean (SD) | Intentional Mean (SD) | Status-Seeking Mean (SD) | Accidental Mean (SD) |
| --- | --- | --- | --- | --- | --- | --- |
| Policy Support | 4.32(1.15) | 0.87[0.86,0.88] | 4.22(1.12) | 4.53(1.15) | 4.23(1.17) | 4.31(1.14) |
| Collective Action Intention | 3.51(1.59) | 0.86[0.84,0.87] | 3.38(1.56) | 3.76(1.58) | 3.52(1.65) | 3.39(1.55) |
| Individual Action Intention | 3.46(1.65) | 0.80[0.77,0.82] | 3.33(1.68) | 3.67(1.62) | 3.38(1.64) | 3.48(1.63) |
| Effectiveness | 3.50(1.45) | 0.89[0.88,0.90] | 3.28(1.23) | 4.11(1.49) | 2.99(1.42) | 3.60(1.41) |
| Identification | 4.16(1.31) | 0.80[0.82,0.84] | 4.08(1.19) | 4.45(1.32) | 3.76(1.33) | 4.33(1.28) |
| Donation | 0.48(0.37) | NA | 0.46(0.38) | 0.48(0.36) | 0.49(0.36) | 0.47(0.37) |

1. **Analytic Procedure**

Our analytic procedure was determined a-priori and pre-registered. It comprised:

**4.1** **primary analyses**: A one-way ANOVA testing main effect of condition on each outcome variables and follow-up pairwise comparisons.

**4.2 Additional robustness analysis**: we conducted Ordinary Least Squares Regression testing the effect of each story (relative to the control) on the outcome variable, when accounting for relevant covariates.

**4.3 Exploratory analysis:** we tested if identification with the protagonist mediated the effect of condition (pairwise comparison) on outcome variable using a simple mediation model.

1. **Impact of Condition on Policy Support**
   1. **ANOVA and Pairwise Comparison**

A one-way ANOVA found a significant main effect of condition on policy support, F (3,899) =3.61, MS=4.73, *p*=0.01. Further pairwise comparisons (using LSD and Bonferroni Corrections) showed , those who read the intentional environmentalist story reported greater support for climate policies, relative to those who read the control; *t*(448)=2.89, *p*=0.004 (Bonferroni corrected p=0.004), *d*=0.27, 95%*CI*[0.09, 0.46], status-seeking environmentalist; *t*(452)=2.78, *p*=0.006 (Bonferroni corrected p=0.01), *d*=0.26, 95%*CI*[0.08, 0.45] and accidental environmentalist ; *t*(453)=2.02, *p*=0.04 (Bonferroni corrected p=0.04), *d*=0.19, 95%*CI*[0.004, 0.37]stories.

Contrastingly, the effect of the status-seeking environmentalist story did not differ significantly from the control, *t*(446)=0.06, *p*=0.95 (Bonferroni corrected p=0.95), *d*=0.01, 95%*CI*[-0.18, 0.19] and accidental stories, *t*(450)=-0.79, *p*=0.427(Bonferroni corrected p=0.43), *d*=-0.07, 95%*CI*[-0.26, 0.11]. Moreover, the effect of the accidental story also did not differ from the control story, *t*(447)=0.87, *p*=0.38(Bonferroni corrected p=0.38), *d*=0.08, 95%*CI*[-0.10, 0.27].

Note: All means and SDs for sample and specific conditions can be found in Table S2.

- 1. **Regression Model including Covariates**

Relative to the control condition, the intentional story (b=0.25, se=0.10, t=2.55, p=0.01), but not the status-seeking (b=-0.01, se=0.10, t=-0.10, p=0.92) and accidental stories (b=0.08, se=0.10, t=0.82, p=0.41) , enhanced policy support, when controlling for relevant covariates (See Fig. S2)

**Fig S2: Regression model testing effects on policy support**


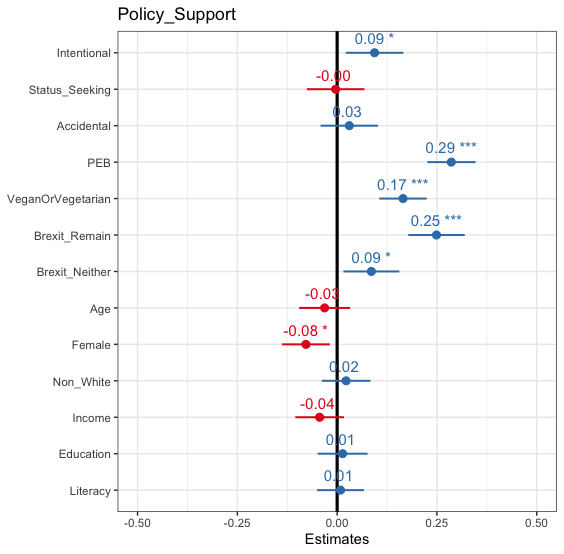


Note: (i) * *p<*0.05, ** *p<*0.01, *** *p<*0.001; (iii) Estimates are standardised coefficients (iii) Error bars represent 95% confidence; (iv) Blue and red represent positive and negative effects respectively; (v) All outcomes measured using composites, on Likert scales of 1(not at all) to 7(extremely); (vi) Omitted categories: Control group, non-vegetarian/vegan diet, Brexit: leave, male, white; (vii) Scales for ordinal/numerical variables: PEB (Past environmental behaviour)– 0-2 scale, Age– continuous numerical variable, Income– Likert scale of 1(less than £20,000) to 6 (more than £100,000), Education– Likert scale of 1(less than O level) to 10 (Doctorate or other professional degree), Literacy– Likert scale of 1 (no proficiency) to 6 (native/bilingual proficiency)

- 1. **Mediation via Identification**

Since only the intentional environmentalist story was able to enhance policy support relative to other condition (i.e., had a significant total effect), all mediation analyses compared the intentional environmentalist story with other conditions.

**TableS3: Effect of condition on policy support via identification**

| Predictor | Effect on identification | Effect on policy support |
| --- | --- | --- |
| **X= Intentional vs Control, M = Identification, Y = Policy Support** | | |
| Direct Effect | | |
| Intentional (vs control) | 0.36** | 0.20^+^ |
| Identification | NA | 0.40*** |
| Indirect Effect via Identification | | |
| Intentional (vs control) | NA | 0.11 [0.04, 0.19] |
| **X= Intentional vs Status, M = Identification, Y = Policy Support** | | |
| Direct Effect | | |
| Intentional (vs status-seeking) | 0.68*** | 0.03 |
| Identification | NA | 0.40*** |
| Indirect Effect via Identification | | |
| Intentional (vs status-seeking) | NA | 0.27 [0.17, 0.39] |
| **X= Intentional vs Accidental, M = Identification, Y = Policy Support** | | |
| Direct Effect | | |
| Intentional (vs accidental) | 0.12 | 0.17^+^ |
| Identification | NA | 0.40*** |
| Indirect Effect via Identification | | |
| Intentional (vs accidental) | NA | 0.05[-0.05, 0.14] |

Note: Mediation analysis conducted using PROCESS model 4; X = Independent variable (predictor), M = mediator, Y = Outcome variable. ***p<0.001, **p<0.01, *p<0.05, ^+^p<0.10. Brackets indicate lower and upper limit estimates derived using 5000 bootstrap confidence intervals.

1. **Impact of Condition on Collective Action Intention**

**6.1 ANOVA and Pairwise Comparison**

A one-way ANOVA found a significant main effect of condition on collective action intentions, F (3,899) =2.85, MS=7.17, *p*=0.03. Further pairwise comparisons (using LSD and Bonferroni Corrections) showed that those who read the intentional environmentalist story reported greater support for climate policies, relative to those who read the control; *t*(448)=2.56, *p*=0.01 (Bonferroni corrected p=0.011), *d*=0.24, 95%*CI*[0.05, 0.43], and accidental environmentalist ; *t*(453)=2.56, *p*=0.01 (Bonferroni corrected p=0.011), *d*=0.24, 95%*CI*[0.05, 0.42]stories. However, the difference between intentional and status-seeking stories was not significant, *t*(451)=1.59, *p*=0.11, (Bonferroni corrected p=0.12) *d*=0.15, 95%*CI*[-0.04, 0.33].

Contrastingly, the effect of the status-seeking environmentalist story did not differ significantly from the control, *t*(445)=0.91, *p*=0.36 (Bonferroni corrected p=0.37), *d*=0.09, 95%*CI*[-0.10, 0.27] and accidental stories, *t*(449)=0.89, *p*=0.37 (Bonferroni corrected p=0.37), *d*=0.08, 95%*CI*[-0.10, 0.27]. Moreover, the effect of the accidental story also did not differ from the control story, *t*(447)=-0.02, *p*=0.98 (Bonferroni corrected p=0.99), *d*=-0.002, 95%*CI*[-0.19, 0.18].

Note: All means and SDs for sample and specific condition can be found in Table S2.

- 1. **Regression Model including Covariates**

Relative to the control condition, the intentional story (b=0.26, se=0.13, t=2.04, p=0.04), but not the status-seeking (b=0.11, se=0.13, t=0.84, p=0.40) and accidental stories (b=-0.01, se=0.12, t=-0.05, p=0.96), enhanced collective action intentions, when controlling for relevant covariates (See Fig. S3)

**Fig S3: Regression model testing effects on Collective Action Intentions**


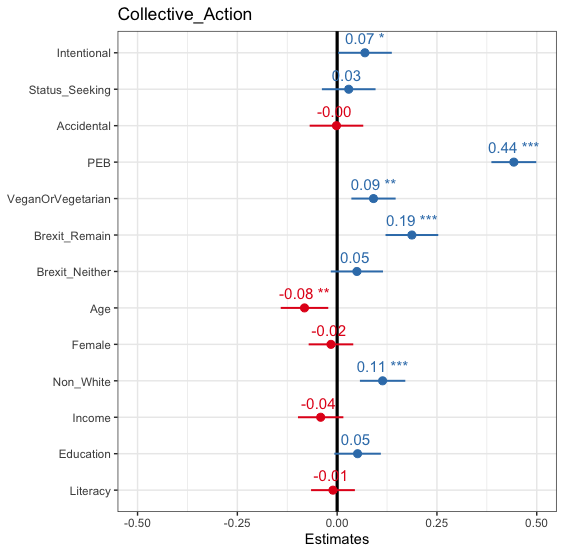


Note: (i) * *p<*0.05, ** *p<*0.01, *** *p<*0.001; (iii) Estimates are standardised coefficients (iii) Error bars represent 95% confidence; (iv) Blue and red represent positive and negative effects respectively; (v) All outcomes measured using composites, on Likert scales of 1(not at all) to 7(extremely); (vi) Omitted categories: Control group, non-vegetarian/vegan diet, Brexit: leave, male, white; (vii) Scales for ordinal/numerical variables: PEB (Past environmental behaviour)– 0-2 scale, Age– continuous numerical variable, Income– Likert scale of 1(less than £20,000) to 6 (more than £100,000), Education– Likert scale of 1(less than O level) to 10 (Doctorate or other professional degree), Literacy– Likert scale of 1 (no proficiency) to 6 (native/bilingual proficiency.

- 1. **Mediation via Identification**

Mediation analyses were conducted only to investigate significant pairwise effects (i.e., where there was a total effect as shown by pairwise comparisons above).

**TableS4: Effect of condition on collective action intentions via identification**

| Predictor | Effect on identification | Effect on collective action intention |
| --- | --- | --- |
| **X= Intentional vs Control, M = Identification, Y = Collective action intention** | | |
| Direct Effect | | |
| Intentional (vs control) | 0.36** | 0.24^+^ |
| Identification | NA | 0.39*** |
| Indirect Effect via Identification | | |
| Intentional (vs control) | NA | 0.14 [0.04, 0.25] |
| **X= Intentional vs Accidental, M = Identification, Y = Collective action intention** | | |
| Direct Effect | | |
| Intentional (vs accidental) | 0.12 | 0.31* |
| Identification | NA | 0.54*** |
| Indirect Effect via Identification | | |
| Intentional (vs accidental) | NA | 0.06 [-0.06, 0.20] |

Note: Mediation analysis conducted using PROCESS model 4; X = Independent variable (predictor), M = mediator, Y = Outcome variable. ***p<0.001, **p<0.01, *p<0.05, ^+^p<0.10. Brackets indicate lower and upper limit estimates derived using 5000 bootstrap confidence intervals.

1. **Impact of Condition on Individual Action Intentions**

**7.1 ANOVA and Pairwise Comparison**

A one-way ANOVA found a that there was no significant main effect of condition on individual action intentions, F (3,899) =1.85, MS=5.00, *p*=0.13. However, as pre-registered in our primary analyses, we conducted further pairwise comparisons (using LSD and Bonferroni Corrections). We found that those who read the intentional environmentalist story reported significantly stronger individual action intentions, relative to those who read the control; t(446)=2.17, p=0.03 (Bonferroni corrected p =0.03), d=0.20, 95%CI[0.02, 0.39)], and marginally stronger individual action intentions that those who read the status-seeking environmentalist story ; t(452)=1.86, p=0.06 (Bonferroni corrected = 0.06), d=0.17, 95%CI[0.01, 0.36] stories. However, the difference between intentional and accidental stories was not significant *t*(453)=1.22, *p*=0.22 (Bonferroni corrected = 0.22), *d*=0.11, 95%*CI*[-0.07, 0.30].

Contrastingly, the effect of the status-seeking environmentalist story did not differ significantly from the control, *t*(445)=0.33, *p*=0.73 (Bonferroni corrected p=0.73), *d*=0.03, 95%*CI*[-0.15, 0.22] and accidental stories, *t*(451)=-0.64, *p*=0.52 (Bonferroni corrected p=0.52), *d*=-0.06, 95%*CI*[-0.24, 0.12]. Moreover, the effect of the accidental story also did not differ from the control story, *t*(446)=0.97, *p*=0.33 (Bonferroni corrected p=0.33), *d*=0.09, 95%*CI*[-0.09, 0.28].

Note: All means and SDs for sample and specific condition can be found in Table S2.

- 1. **Regression Model including Covariates**

Relative to the control condition, the intentional story (b=0.25, se=0.14, t=1.18, p=0.07), but not status-seeking (b=0.09, se=0.14, t=0.65, p=0.52) and accidental stories (b=0.17, se=0.13, t=1.26, p=0.21), marginally significantly enhanced individual action intentions, when controlling for relevant covariates (See Fig. S4)

**Fig S4: Regression model testing effects on Individual Action Intentions**


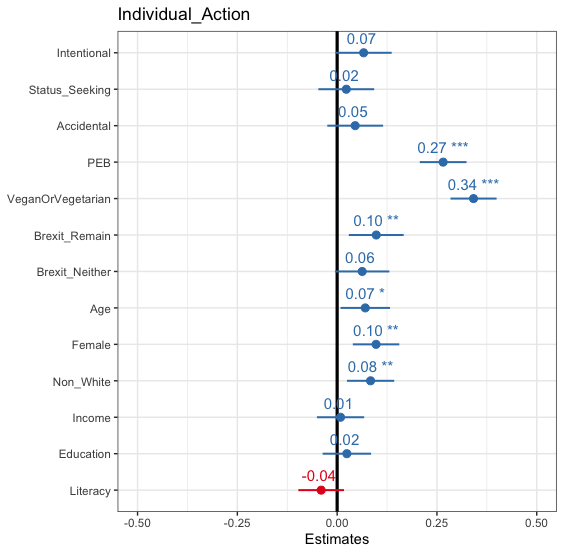


Note: (i) * *p<*0.05, ** *p<*0.01, *** *p<*0.001; (iii) Estimates are standardised coefficients (iii) Error bars represent 95% confidence; (iv) Blue and red represent positive and negative effects respectively; (v) All outcomes measured using composites, on Likert scales of 1(not at all) to 7(extremely); (vi) Omitted categories: Control group, non-vegetarian/vegan diet, Brexit: leave, male, white; (vii) Scales for ordinal/numerical variables: PEB (Past environmental behaviour)– 0-2 scale, Age– continuous numerical variable, Income– Likert scale of 1(less than £20,000) to 6 (more than £100,000), Education– Likert scale of 1(less than O level) to 10 (Doctorate or other professional degree), Literacy– Likert scale of 1 (no proficiency) to 6 (native/bilingual proficiency.

- 1. **Mediation via Identification**

Mediation analyses were conducted only to investigate significant pairwise effects (i.e., where there was a total effect as shown by pairwise comparisons above).

**TableS5: Effect of condition on individual action intentions via identification**

| Predictor | Effect on identification | Effect on individual action intentions |
| --- | --- | --- |
| **X= Intentional vs Control, M = Identification, Y = Individual action intentions** | | |
| Direct Effect | | |
| Intentional (vs control) | 0.36** | 0.20 |
| Identification | NA | 0.39*** |
| Indirect Effect via Identification | | |
| Intentional (vs control) | NA | 0.14 [0.05, 0.24] |
| **X= Intentional vs Status, M = Identification, Y = Individual action intentions** | | |
| Direct Effect | | |
| Intentional (vs status-seeking) | 0.68*** | -0.04 |
| Identification | NA | 0.47*** |
| Indirect Effect via Identification | | |
| Intentional (vs status-seeking) | NA | 0.32 [0.19, 0.46] |

Note: Mediation analysis conducted using PROCESS model 4; X = Independent variable (predictor), M = mediator, Y = Outcome variable. ***p<0.001, **p<0.01, *p<0.05, ^+^p<0.10. Brackets indicate lower and upper limit estimates derived using 5000 bootstrap confidence intervals.

**8. Impact of Condition on Perceived Effectiveness of Character**

- 1. **ANOVA and Pairwise Comparison**

A One-way ANOVA found a main effect of condition on perceived effectiveness of the character, F (3,899) =26.93, MS=52.25, *p*<0.001. Further pair-wise comparisons (with LSD and Bonferroni corrections found that), those who read the intentional environmentalist story perceived George to be significantly more effective than did those who read the control *t*(437)=6.46, *p*<0.001 (Bonferroni corrected p<0.001), *d*=0.60, 95%*CI*[0.41, 0.80], status-seeking *t*(451)=8.19, *p*<0.001 (Bonferroni corrected p<0.001), *d*=0.77, 95%*CI*[0.57, 0.97], and accidental stories, *t*(452)=3.75, *p*<0.001 (Bonferroni corrected p<0.001), *d*=0.35, 95%*CI*[0.16, 0.54].

Moreover, those who read the status-seeking environmentalist story perceived George to be significantly less effective than did those who read the control *t*(439)=-2.30, *p*=0.02 (Bonferroni corrected p=0.02), *d*=-0.22, 95%*CI*[-0.40, -0.03], and accidental stories *t*(451)=-4.58, *p*<0.001 (Bonferroni corrected p<0.001), *d*=-0.43, 95%*CI*[-0.62, -0.24].

Finally, those who read the accidental environmentalist story perceived George to be significantly more effective than did those who read the control story, *t*(441)=2.57, *p*=0.01 (Bonferroni corrected p=0.011), *d*=0.24, 95%*CI*[0.05, 0.43].

**8.2 Regression Model including Covariates.**

Relative to the control condition, the intentional story (b=0.76, se=0.13, t=5.90, p<0.001) and accidental story (b=0.31, se=0.13, t=2.42, p=0.02) enhanced effectiveness, whereas the status seeking story reduced effectiveness (b=-0.30, se=0.13, t=-2.37, p=0.02), when controlling for relevant covariates (See Fig. S5)

**Fig S5: Regression model testing effects on Character’s Effectiveness**


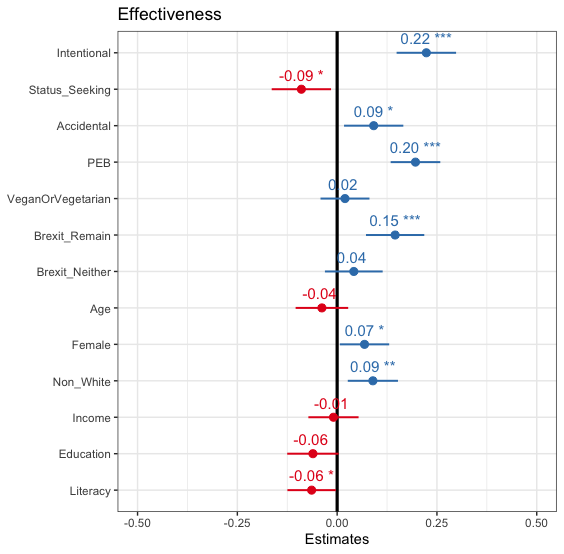


Note: (i) * *p<*0.05, ** *p<*0.01, *** *p<*0.001; (iii) Estimates are standardised coefficients (iii) Error bars represent 95% confidence; (iv) Blue and red represent positive and negative effects respectively; (v) All outcomes measured using composites, on Likert scales of 1(not at all) to 7(extremely); (vi) Omitted categories: Control group, non-vegetarian/vegan diet, Brexit: leave, male, white; (vii) Scales for ordinal/numerical variables: PEB (Past environmental behaviour)– 0-2 scale, Age– continuous numerical variable, Income– Likert scale of 1(less than £20,000) to 6 (more than £100,000), Education– Likert scale of 1(less than O level) to 10 (Doctorate or other professional degree), Literacy– Likert scale of 1 (no proficiency) to 6 (native/bilingual proficiency.

- 1. **Mediation via Identification**

Mediation analyses were conducted only to investigate significant pairwise effects (i.e., where there was a total effect as shown by pairwise comparisons above). Moreover, since the mediation analysis was conducted to understand the effect of the intentional environmentalist story, only comparisons with intentional environmentalist story are added as predictors.

**TableS6: Effect of condition on effectiveness via identification**

| Predictor | Effect on identification | Effect on effectiveness |
| --- | --- | --- |
| **X= Intentional vs Control, M = Identification, Y = Effectiveness** | | |
| Direct Effect | | |
| Intentional (vs control) | 0.36** | 0.63*** |
| Identification | NA | 0.54*** |
| Indirect Effect via Identification | | |
| Intentional (vs control) | NA | 0.20[0.07, 0.33] |
| **X= Intentional vs Status, M = Identification, Y = Effectiveness** | | |
| Direct Effect | | |
| Intentional (vs status-seeking) | 0.68*** | 0.67*** |
| Identification | NA | 0.66*** |
| Indirect Effect via Identification | | |
| Intentional (vs status-seeking) | NA | 0.45 [0.29, 0.62] |
| **X= Intentional vs Accidental, M = Identification, Y = Effectiveness** | | |
| Direct Effect | | |
| Intentional (vs accidental) | 0.12 | 0.43*** |
| Identification | NA | 0.68*** |
| Indirect Effect via Identification | | |
| Intentional (vs accidental) | NA | 0.08[-0.08,0.24] |

Note: Mediation analysis conducted using PROCESS model 4; X = Independent variable (predictor), M = mediator, Y = Outcome variable. ***p<0.001, **p<0.01, *p<0.05, ^+^p<0.10. Brackets indicate lower and upper limit estimates derived using 5000 bootstrap confidence intervals.

**9. Impact of Condition on Donation Amount**

**9.1** **ANOVA and Pairwise Comparison**

There was no significant main effect of condition on donation amount, F (3,899) =0.41, MS=0.06, *p*=0.747. However, as pre-registered in our primary analyses, we conducted further pairwise comparisons (using LSD and Bonferroni Corrections). We found no significant pairwise differences:

- intentional vs control: *t*(446)=-0.80, *p*=0.43 (Bonferroni corrected p=0.43), *d*=-0.08, 95%*CI*[-0.26, 0.11]
- intentional vs. status-seeking: *t*(452)=0.24, *p*=0.81 (Bonferroni corrected p=0.81), *d*=0.02, 95%*CI*[-0.16, 0.21]
- intentional vs accidental: *t*(453)=-0.37, *p*=0.71 (Bonferroni corrected p=0.71), *d*=-0.03, 95%*CI*[-0.22, 0.15]
- status-seeking vs. control: *t*(445)=1.03, *p*=0.30 (Bonferroni corrected p=0.31), *d*=0.10, 95%*CI*[-0.09, 0.28]
- status-seeking vs accidental: *t*(451)=0.61, *p*=0.54 (Bonferroni corrected p=0.54), *d*=0.06, 95%*CI*[-0.13, 0.24]
- accidental vs control: *t*(446)=0.43, *p*=0.67 (Bonferroni corrected p=0.67), *d*=0.04, 95%*CI*[-0.14, 0.23]

Given non-significant pairwise comparisons, no further analyses were conducted.

**10. Deviation from Pre-registration**

Our study incorporated all our pre-registered primary independent variables and all pre-registered outcome variables except for second-order beliefs. Moreover, we tested all our primary hypotheses except for, “Second order beliefs will mediate the effect of the treatment narrative (vs. control narrative) on pro-environmental outcomes.”

Our story informed participants about the day-in-the life of one person– George. In order to study the influence of a singular protagonist, we eliminated any information readers might receive about George’s social group (see Stimuli).

Upon more in-depth search for literature post pre-registration indicated to us that second-order beliefs were likely to be affected by information people receive about their group at large^5,6^, rather than the information they receive about one individual. Therefore, given its composition, we realised that our story was not crafted to impact second-order beliefs. Instead, our story fleshed out the character and his mental states, likely making him more relatable. Therefore, we decided to explore whether identification with George mediated the effect of condition (this was pre-registered as an exploratory analysis).

**11. References**

1. Thomas, G., Hartley, R. D. & Kincaid, J. P. Test-Retest and Inter-Analyst Reliability of the Automated Readability Index, Flesch Reading Ease Score, and the Fog Count. *J. Read. Behav.* **7**, 149–154 (1975).

2. *The Sixth Carbon Budget The UK’s path to Net Zero*. (2020).

3. Leiserowitz, A. *Climate change in the American mind: November 2019*. (University and George Mason University, 2019).

4. Sabherwal, A. *et al.* The Greta Thunberg Effect: Familiarity with Greta Thunberg predicts intentions to engage in climate activism in the United States. *J. Appl. Soc. Psychol.* (2021) doi:10.1111/jasp.12737.

5. Sabherwal, A., Pearson, A. R. & Sparkman, G. Anger consensus messaging can enhance expectations for collective action and support for climate mitigation. *J. Environ. Psychol.* **76**, 101640 (2021).

6. Mildenberger, M. & Tingley, D. Beliefs about Climate Beliefs: The Importance of Second-Order Opinions for Climate Politics. *Br. J. Polit. Sci.* **49**, 1279–1307 (2019).
